# Bare Limbs

**Published:** 1873-02

**Authors:** 


### BARE LIMBS

Of children gratify the vanity of mothers, but they send multitudes of beautiful children to a premature grave. It would be safer to have the arms, hands, feet, and legs warmly encased in double thickness of woolen flannel, with nothing whatever on the body but a common night-gown, in the fall of the year. It is especially important to keep the extremities of children and infants warm for every second of their existence. Whether a child is sick or well, when the hands and feet begin to get cold, it is nearing the grave, because the blood retreats to the inner organs, oppresses them, causing painful and dangerous congestions and inflammations, which often induce death in a few hours, as in croup, diphtheria, quinsy, and the like. A young mother should never go to bed until she has noticed that the feet of her little sleeping ones are abundantly warm; to be assured of that is to know that croup before the morning is impossible.

